# Supplementary material for: Prevalence of glaucoma in Africa: A systematic review and Bayesian meta-analysis
Source: PLoS One. 2025 Aug 14;20(8):e0330567. doi: 10.1371/journal.pone.0330567 (PMC12352844; doi:10.1371/journal.pone.0330567)
Supplement: S1 Table — (DOCX) [file pone.0330567.s001.docx]

**Web of Science.**

| **#** | **Search Query** | **Results** |
| --- | --- | --- |
| 1 | ALL=(glaucoma) | 91780 |
| 2 | ALL=(prevalence) | 1377848 |
| 3 | ALL=(incidence) | 1124026 |
| 4 | #2 OR #3 | 2377185 |
| 5 | ((ALL=(population)) OR ALL=(survey)) OR ALL=(epidemiology) | 5952319 |
| 6 | ALL=(clinical characteristics) | 735277 |
| 7 | ALL=(africa) | 921818 |
| 8 | #1 AND #4 AND #5 AND #6 AND #7 | 9 |

Date run: 25^th^ January 2025.

**Scopus:** Four Studies**.**

( TITLE-ABS-KEY ( glaucoma ) ) AND ( ( TITLE-ABS-KEY ( prevalence ) OR TITLE-ABS-KEY ( incidence ) ) ) AND ( TITLE-ABS-KEY ( clinical AND characteristics ) ) AND ( TITLE-ABS-KEY ( africa ) )

Date run: 25^th^ January 2025.

**PubMed.**

| **Search number** | **Query** | **Search Details** | **Results** |
| --- | --- | --- | --- |
| 11 | ((((glaucoma) AND ((prevalence) OR (incidence))) AND (((population) OR (survey)) OR (epidemiology))) AND (clinical characteristics)) AND (Africa) | ("glaucoma"[MeSH Terms] OR "glaucoma"[All Fields] OR "glaucomas"[All Fields] OR "glaucoma s"[All Fields]) AND ("epidemiology"[MeSH Subheading] OR "epidemiology"[All Fields] OR "prevalence"[All Fields] OR "prevalence"[MeSH Terms] OR "prevalance"[All Fields] OR "prevalences"[All Fields] OR "prevalence s"[All Fields] OR "prevalent"[All Fields] OR "prevalently"[All Fields] OR "prevalents"[All Fields] OR ("epidemiology"[MeSH Subheading] OR "epidemiology"[All Fields] OR "incidence"[All Fields] OR "incidence"[MeSH Terms] OR "incidences"[All Fields] OR "incident"[All Fields] OR "incidents"[All Fields])) AND ("populate"[All Fields] OR "populated"[All Fields] OR "populates"[All Fields] OR "populating"[All Fields] OR "population"[MeSH Terms] OR "population"[All Fields] OR "population groups"[MeSH Terms] OR ("population"[All Fields] AND "groups"[All Fields]) OR "population groups"[All Fields] OR "populations"[All Fields] OR "population s"[All Fields] OR "populational"[All Fields] OR "populous"[All Fields] OR ("survey s"[All Fields] OR "surveyed"[All Fields] OR "surveying"[All Fields] OR "surveys and questionnaires"[MeSH Terms] OR ("surveys"[All Fields] AND "questionnaires"[All Fields]) OR "surveys and questionnaires"[All Fields] OR "survey"[All Fields] OR "surveys"[All Fields]) OR ("epidemiologies"[All Fields] OR "epidemiology"[MeSH Subheading] OR "epidemiology"[All Fields] OR "epidemiology"[MeSH Terms] OR "epidemiology s"[All Fields])) AND (("ambulatory care facilities"[MeSH Terms] OR ("ambulatory"[All Fields] AND "care"[All Fields] AND "facilities"[All Fields]) OR "ambulatory care facilities"[All Fields] OR "clinic"[All Fields] OR "clinic s"[All Fields] OR "clinical"[All Fields] OR "clinically"[All Fields] OR "clinicals"[All Fields] OR "clinics"[All Fields]) AND ("characteristic"[All Fields] OR "characteristics"[All Fields])) AND ("africa"[MeSH Terms] OR "africa"[All Fields] OR "africa s"[All Fields] OR "africas"[All Fields]) | 34 |
| 10 | Africa | "africa"[MeSH Terms] OR "africa"[All Fields] OR "africa s"[All Fields] OR "africas"[All Fields] | 517,023 |
| 9 | clinical characteristics | ("ambulatory care facilities"[MeSH Terms] OR ("ambulatory"[All Fields] AND "care"[All Fields] AND "facilities"[All Fields]) OR "ambulatory care facilities"[All Fields] OR "clinic"[All Fields] OR "clinic s"[All Fields] OR "clinical"[All Fields] OR "clinically"[All Fields] OR "clinicals"[All Fields] OR "clinics"[All Fields]) AND ("characteristic"[All Fields] OR "characteristics"[All Fields]) | 716,789 |
| 8 | ((population) OR (survey)) OR (epidemiology) | "populate"[All Fields] OR "populated"[All Fields] OR "populates"[All Fields] OR "populating"[All Fields] OR "population"[MeSH Terms] OR "population"[All Fields] OR "population groups"[MeSH Terms] OR ("population"[All Fields] AND "groups"[All Fields]) OR "population groups"[All Fields] OR "populations"[All Fields] OR "population s"[All Fields] OR "populational"[All Fields] OR "populous"[All Fields] OR ("survey s"[All Fields] OR "surveyed"[All Fields] OR "surveying"[All Fields] OR "surveys and questionnaires"[MeSH Terms] OR ("surveys"[All Fields] AND "questionnaires"[All Fields]) OR "surveys and questionnaires"[All Fields] OR "survey"[All Fields] OR "surveys"[All Fields]) OR ("epidemiologies"[All Fields] OR "epidemiology"[MeSH Subheading] OR "epidemiology"[All Fields] OR "epidemiology"[MeSH Terms] OR "epidemiology s"[All Fields]) | 6,476,458 |
| 7 | epidemiology | "epidemiologies"[All Fields] OR "epidemiology"[MeSH Subheading] OR "epidemiology"[All Fields] OR "epidemiology"[MeSH Terms] OR "epidemiology s"[All Fields] | 3,364,584 |
| 6 | survey | "survey s"[All Fields] OR "surveyed"[All Fields] OR "surveying"[All Fields] OR "surveys and questionnaires"[MeSH Terms] OR ("surveys"[All Fields] AND "questionnaires"[All Fields]) OR "surveys and questionnaires"[All Fields] OR "survey"[All Fields] OR "surveys"[All Fields] | 1,966,698 |
| 5 | population | "populate"[All Fields] OR "populated"[All Fields] OR "populates"[All Fields] OR "populating"[All Fields] OR "population"[MeSH Terms] OR "population"[All Fields] OR "population groups"[MeSH Terms] OR ("population"[All Fields] AND "groups"[All Fields]) OR "population groups"[All Fields] OR "populations"[All Fields] OR "population s"[All Fields] OR "populational"[All Fields] OR "populous"[All Fields] | 2,998,063 |
| 4 | (prevalence) OR (incidence) | "epidemiology"[MeSH Subheading] OR "epidemiology"[All Fields] OR "prevalence"[All Fields] OR "prevalence"[MeSH Terms] OR "prevalance"[All Fields] OR "prevalences"[All Fields] OR "prevalence s"[All Fields] OR "prevalent"[All Fields] OR "prevalently"[All Fields] OR "prevalents"[All Fields] OR "epidemiology"[MeSH Subheading] OR "epidemiology"[All Fields] OR "incidence"[All Fields] OR "incidence"[MeSH Terms] OR "incidences"[All Fields] OR "incident"[All Fields] OR "incidents"[All Fields] | 4,614,880 |
| 3 | incidence | "epidemiology"[MeSH Subheading] OR "epidemiology"[All Fields] OR "incidence"[All Fields] OR "incidence"[MeSH Terms] OR "incidences"[All Fields] OR "incident"[All Fields] OR "incidents"[All Fields] | 4,066,511 |
| 2 | prevalence | "epidemiology"[MeSH Subheading] OR "epidemiology"[All Fields] OR "prevalence"[All Fields] OR "prevalence"[MeSH Terms] OR "prevalance"[All Fields] OR "prevalences"[All Fields] OR "prevalence s"[All Fields] OR "prevalent"[All Fields] OR "prevalently"[All Fields] OR "prevalents"[All Fields] | 3,956,865 |
| 1 | glaucoma | "glaucoma"[MeSH Terms] OR "glaucoma"[All Fields] OR "glaucomas"[All Fields] OR "glaucoma s"[All Fields] | 90,583 |

Date run: 25^th^ January 2025.
