# Supplementary figures and images for: Prevalence of glaucoma in Africa: A systematic review and Bayesian meta-analysis
Source: PLoS One. 2025 Aug 14;20(8):e0330567. doi: 10.1371/journal.pone.0330567 (PMC12352844; doi:10.1371/journal.pone.0330567)

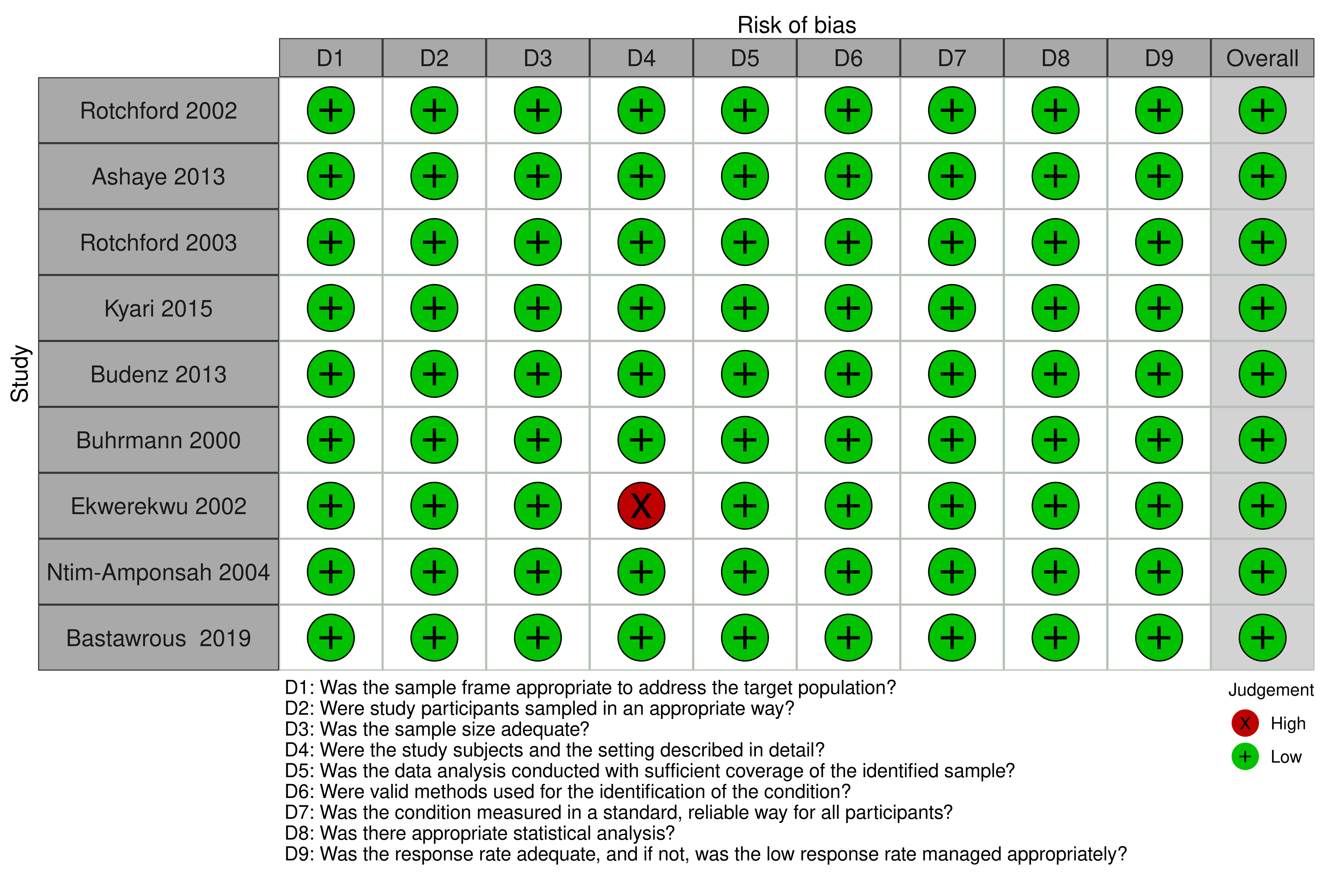

Supplement: S1 Fig — (TIF) [file pone.0330567.s004.tif]
