# Supplementary material for: Prevalence of glaucoma in Africa: A systematic review and Bayesian meta-analysis
Source: PLoS One. 2025 Aug 14;20(8):e0330567. doi: 10.1371/journal.pone.0330567 (PMC12352844; doi:10.1371/journal.pone.0330567)
Supplement: S2 Fig — (PDF) [file pone.0330567.s005.pdf]

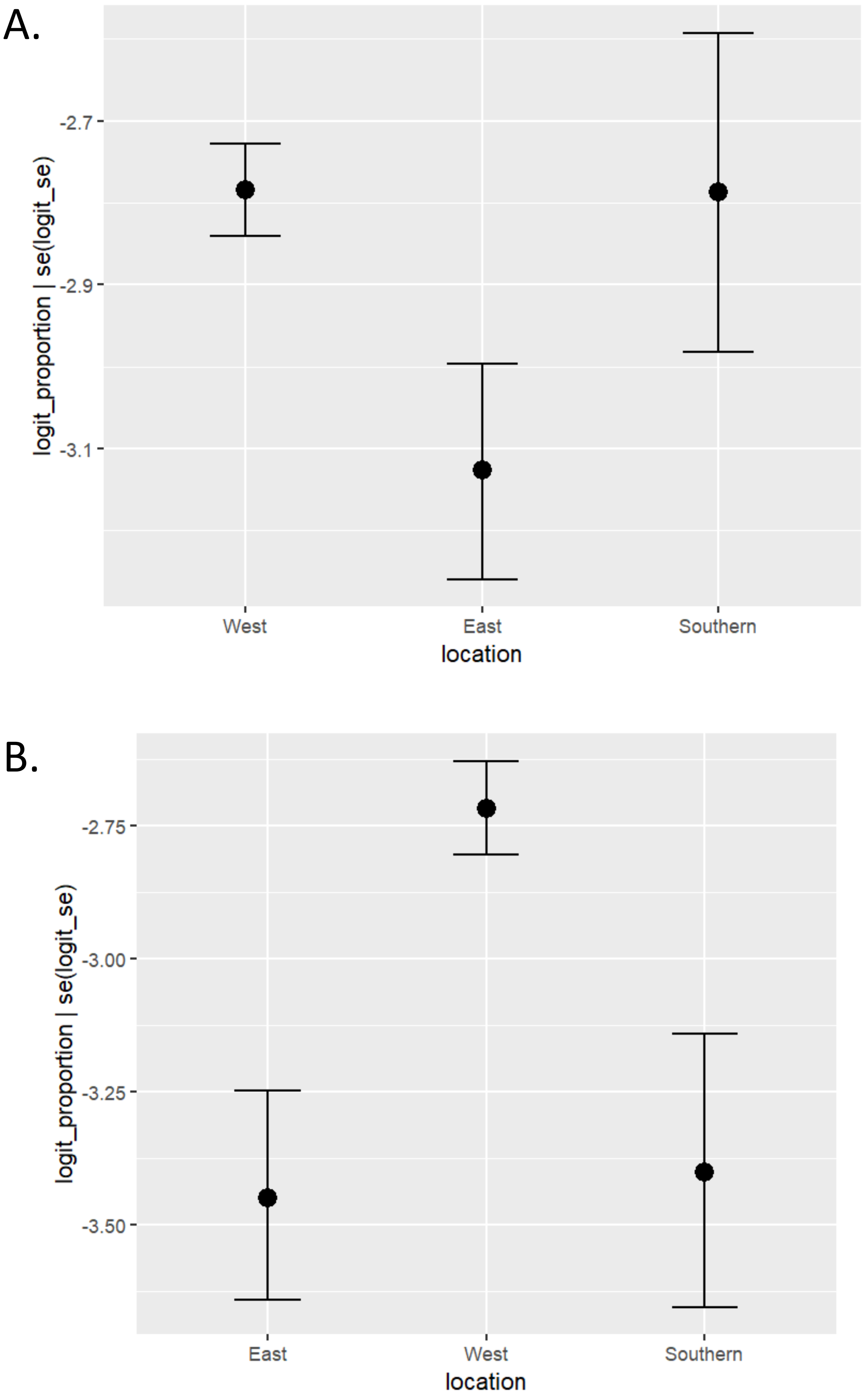

Marginal effects of glaucoma (unclassified) (A) and Primary Open-Angle Glaucoma (B) by geographic location.
