# Supplementary material for: Prevalence of glaucoma in Africa: A systematic review and Bayesian meta-analysis
Source: PLoS One. 2025 Aug 14;20(8):e0330567. doi: 10.1371/journal.pone.0330567 (PMC12352844; doi:10.1371/journal.pone.0330567)
Supplement: S3 Fig — (PDF) [file pone.0330567.s006.pdf]

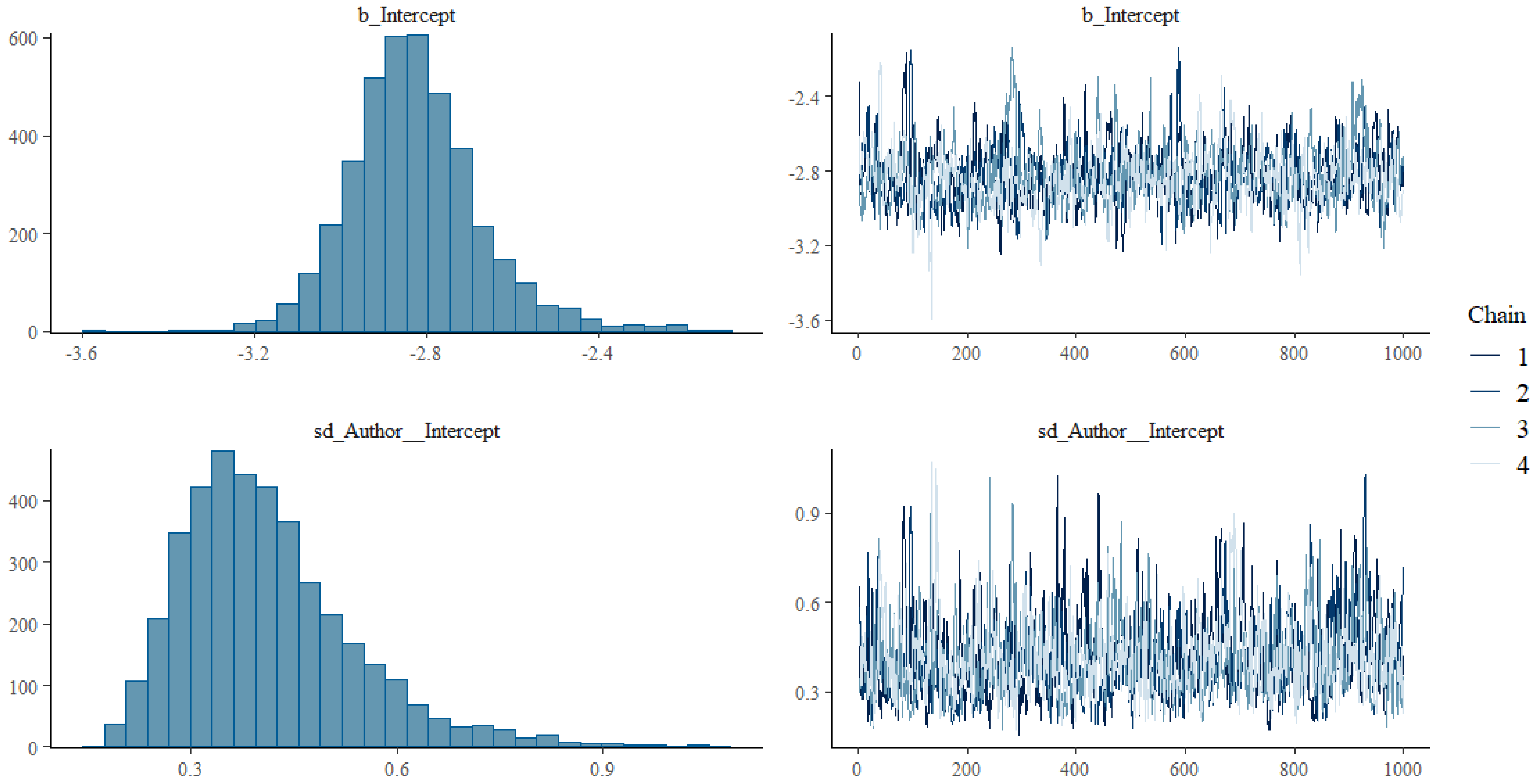

Model diagnostics for glaucoma (unclassified). Values for μ (b_Intercept) in log-odds scale: e.g. -0.94 in log-odds equals to 0.28 in probability scale. On the left: Posterior predictive density plots illustrating the highest density area for predictive values of μ (b_Intercept), and for predictive values of τ (sd_Author__Intercept). On the right: Trace plots, to illustrate model convergence for μ and τ.

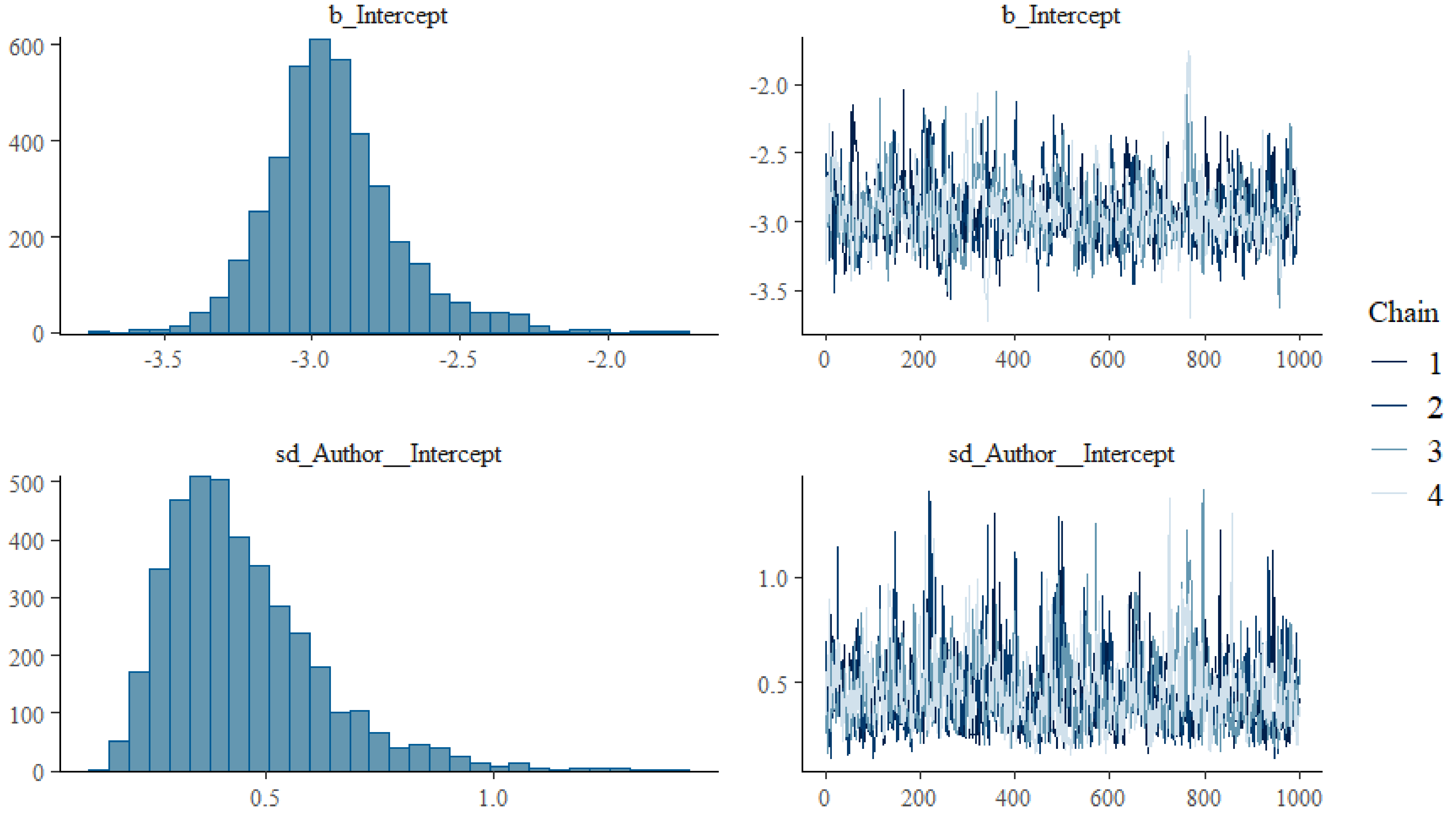

Model diagnostics for Primary Open-Angle Glaucoma. Values for μ (b_Intercept) in log-odds scale: e.g. -0.94 in log-odds equals to 0.28 in probability scale. On the left: Posterior predictive density plots illustrating the highest density area for predictive values of μ (b_Intercept), and for predictive values of τ (sd_Author__Intercept). On the right: Trace plots, to illustrate model convergence for μ and τ.

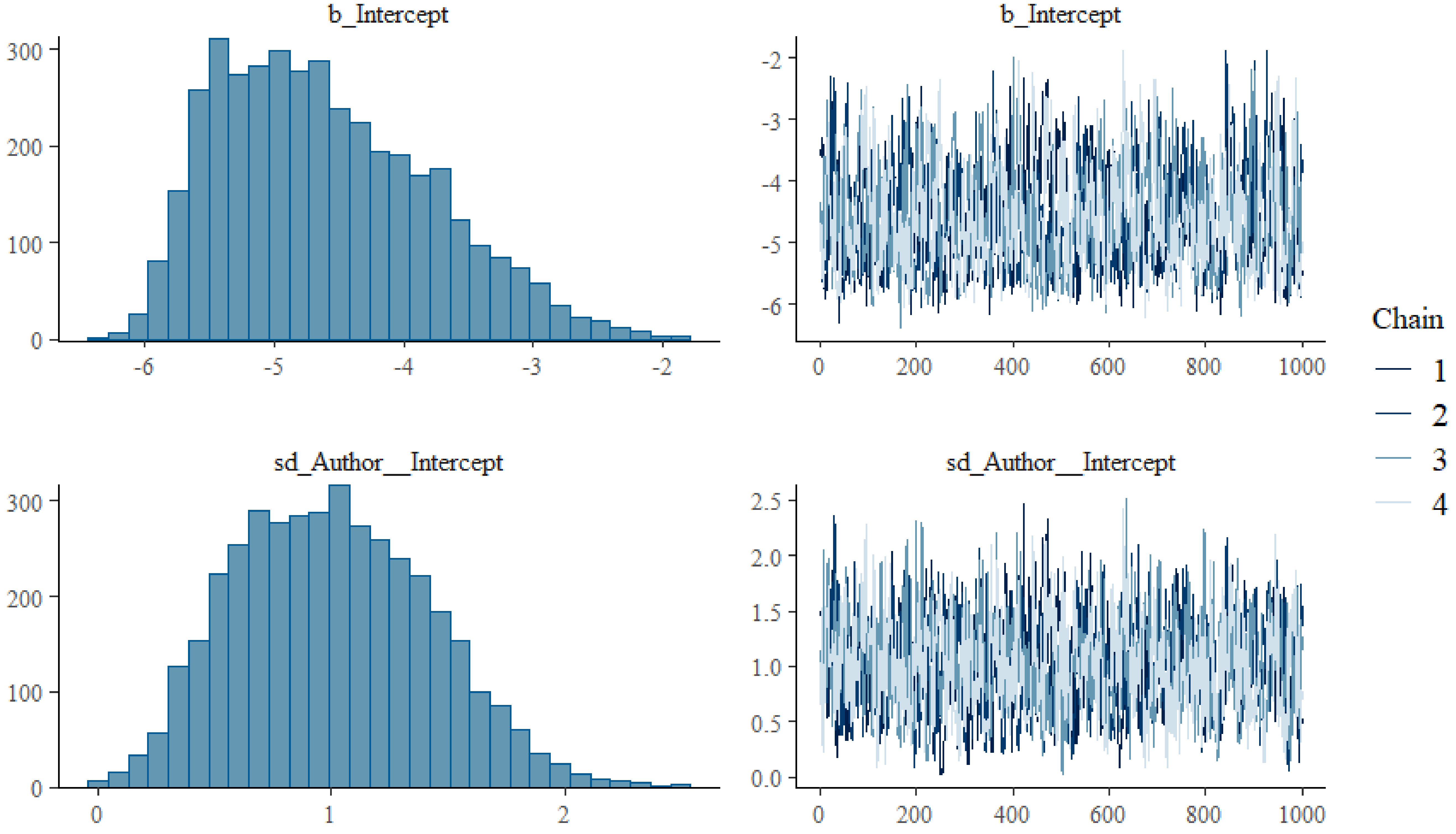

Model diagnostics for Primary Angle-Closure Glaucoma. Values for μ (b_Intercept) in log-odds scale: e.g. -0.94 in log-odds equals to 0.28 in probability scale. On the left: Posterior predictive density plots illustrating the highest density area for predictive values of μ (b_Intercept), and for predictive values of τ (sd_Author__Intercept). On the right: Trace plots, to illustrate model convergence for μ and τ.

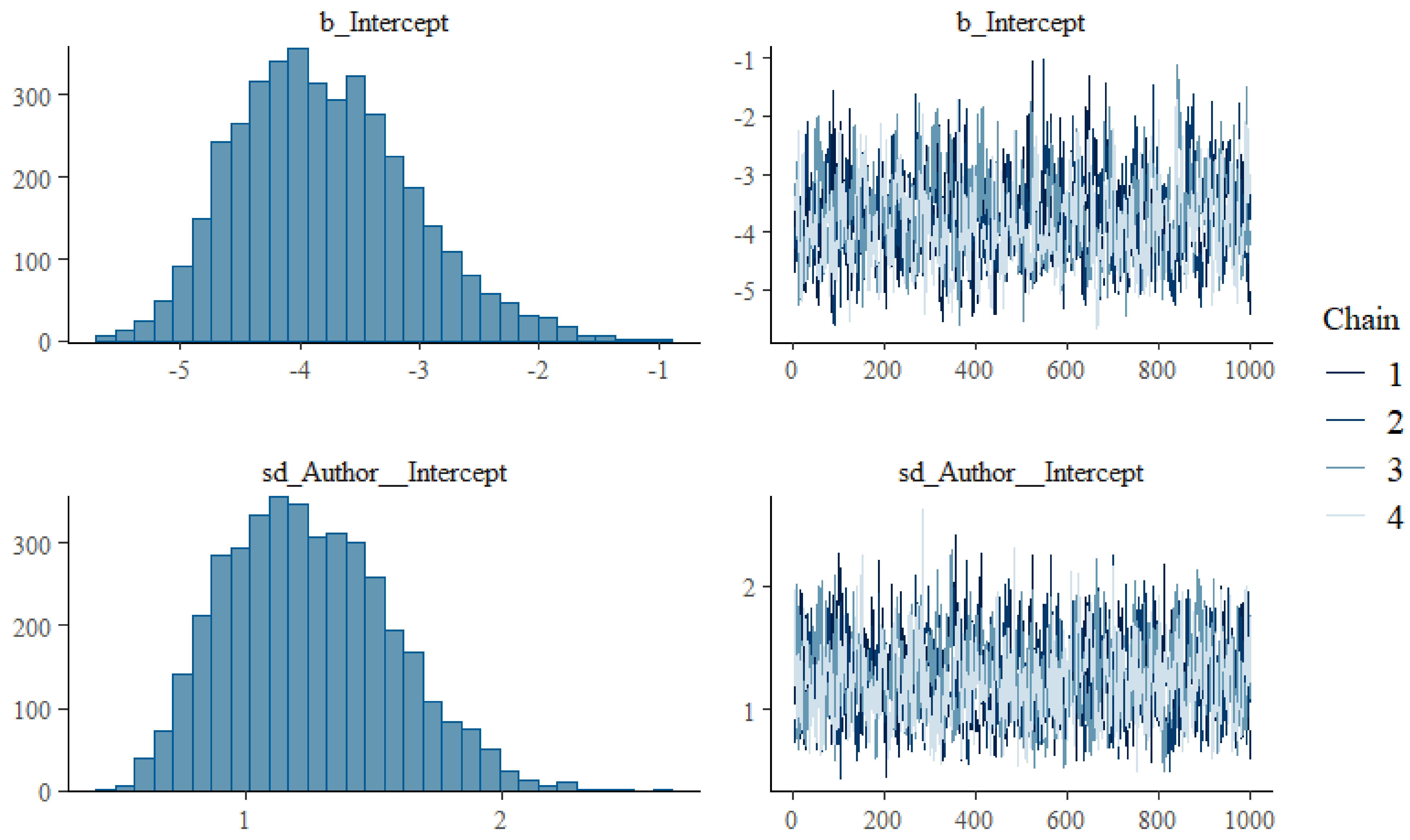

Model diagnostics for secondary glaucoma. Values for μ (b_Intercept) in log-odds scale: e.g. -0.94 in log-odds equals to 0.28 in probability scale. On the left: Posterior predictive density plots illustrating the highest density area for predictive values of μ (b_Intercept), and for predictive values of τ (sd_Author__Intercept). On the right: Trace plots, to illustrate model convergence for μ and τ.

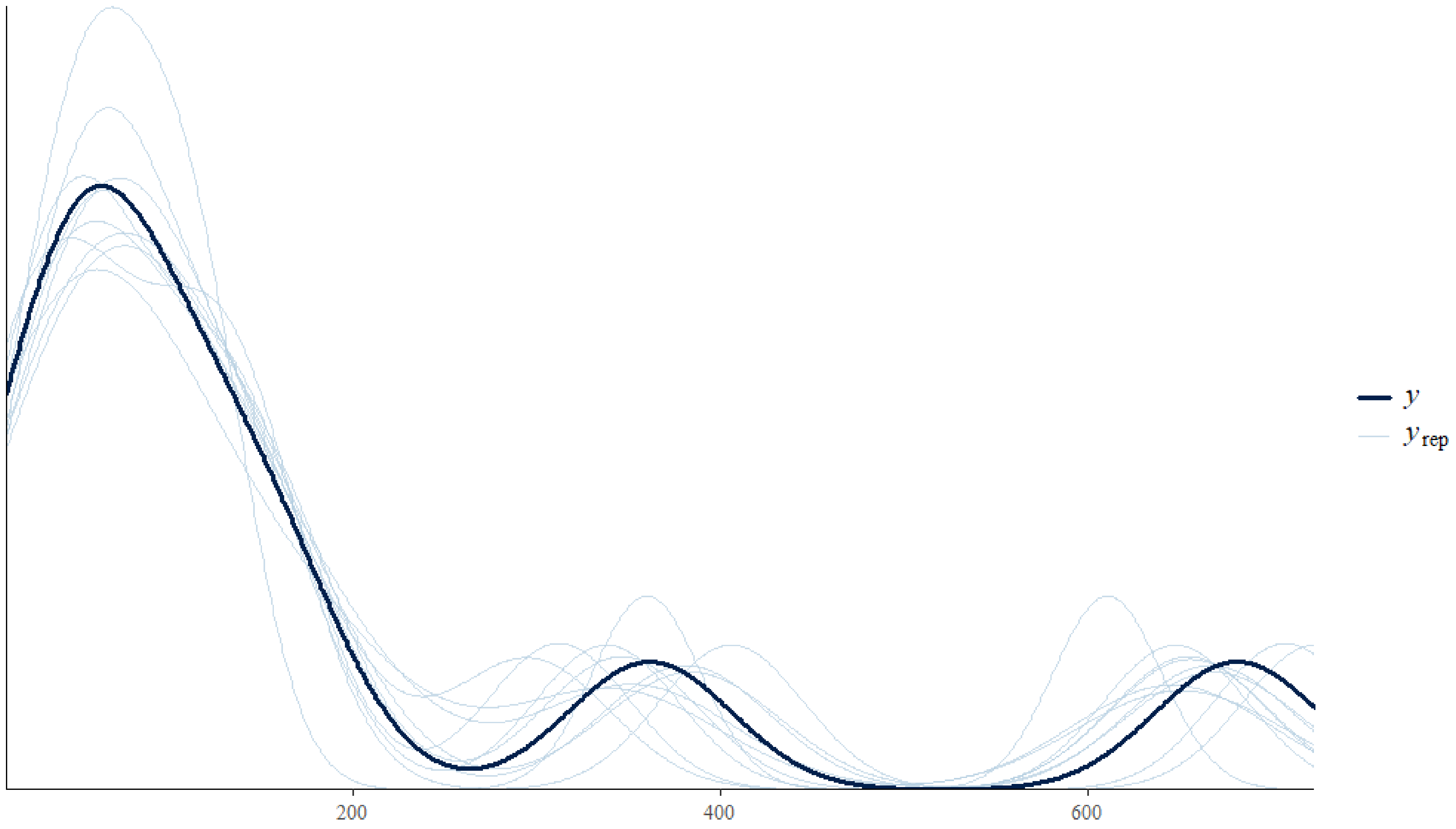

Posterior predictive check (PPcheck) plot for glaucoma (unclassified). Plot illustrating the Bayesian model convergence, showing the comparison between the trajectories of fitted model predictions (lighter lines) and the actual observed data (darker line).

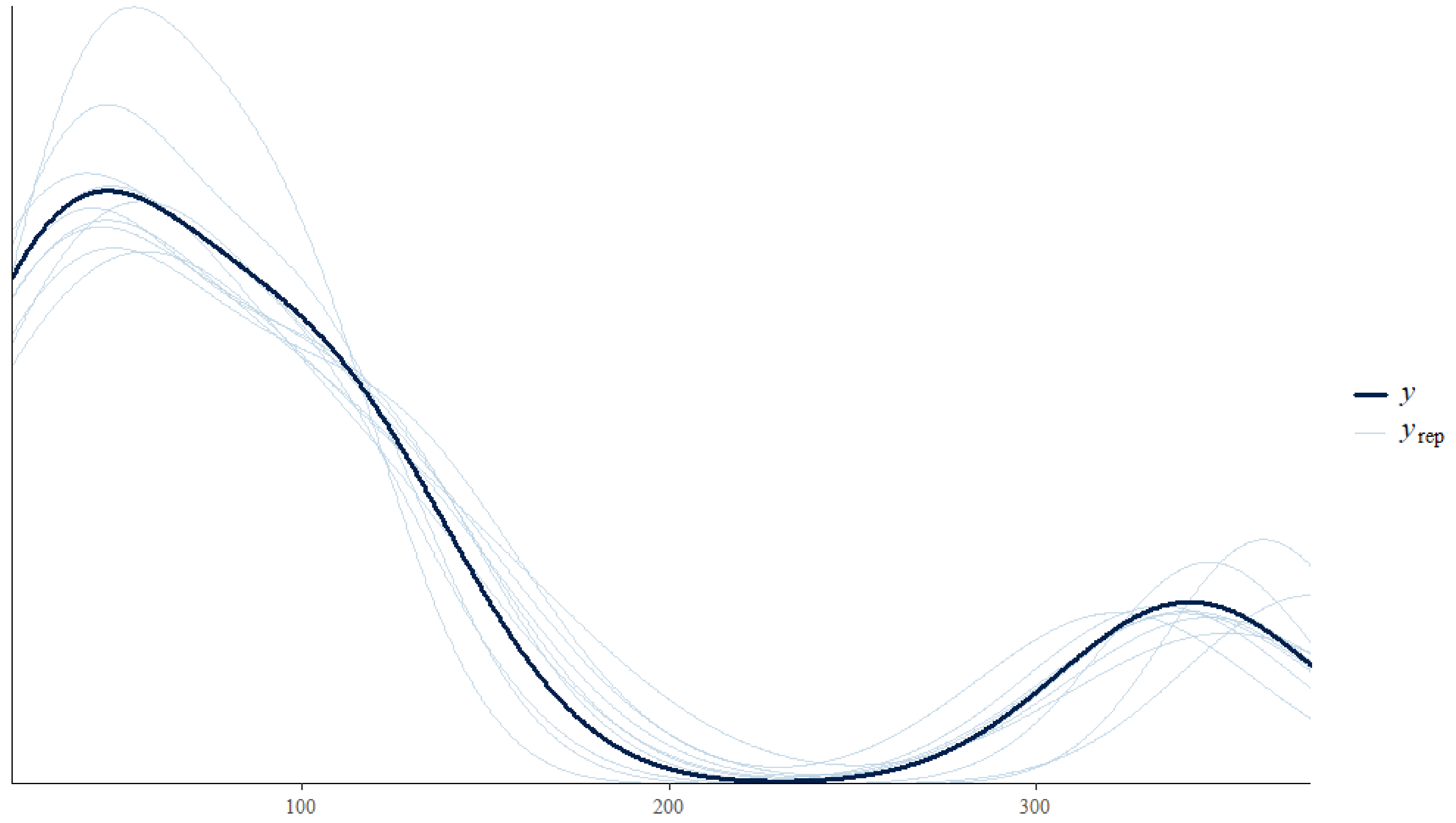

Posterior predictive check (PPcheck) plot for Primary Open-Angle Glaucoma. Plot illustrating the Bayesian model convergence, showing the comparison between the trajectories of fitted model predictions (lighter lines) and the actual observed data (darker line).

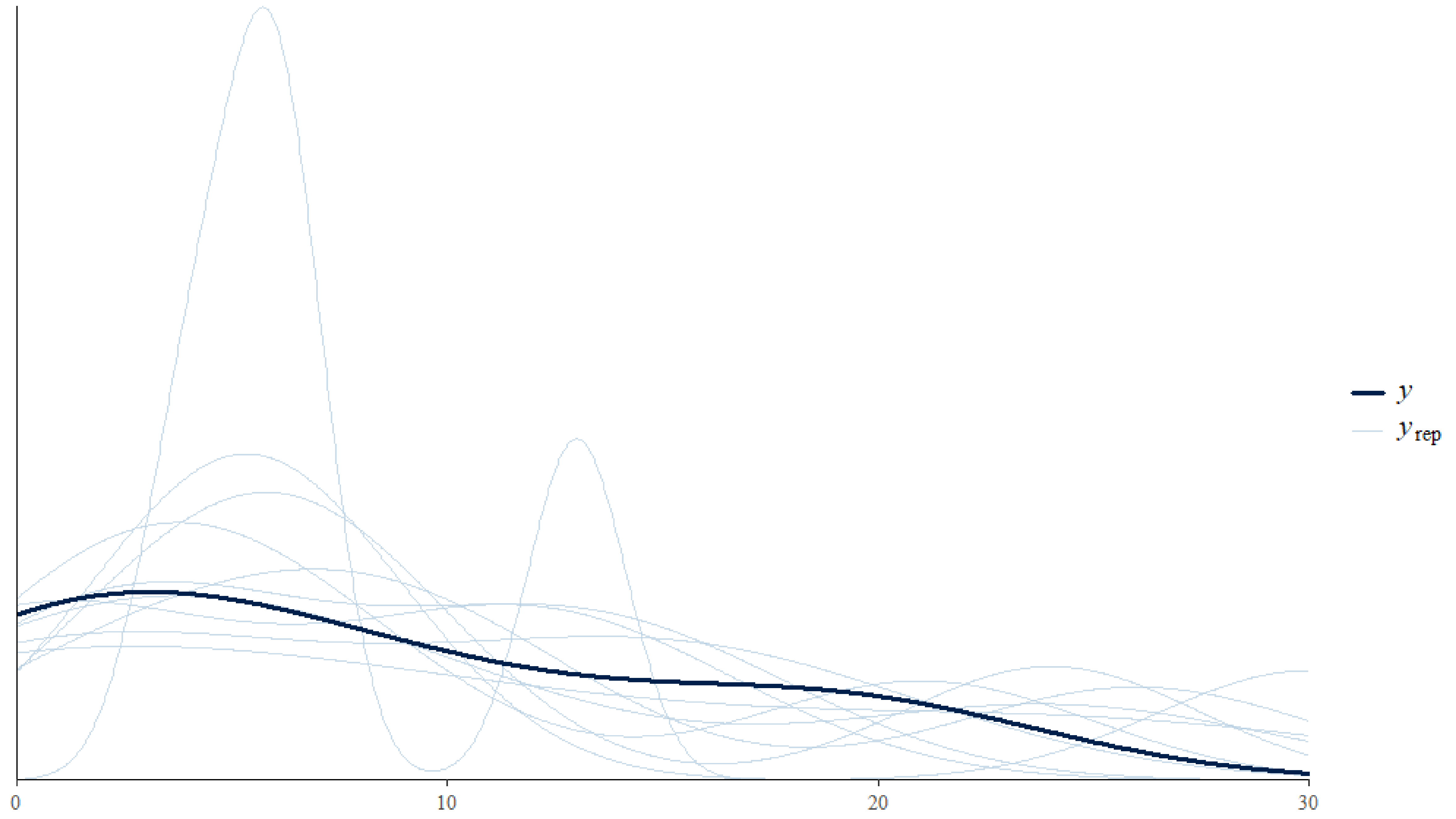

Posterior predictive check (PPcheck) plot for Primary Angle-Closure Glaucoma. Plot illustrates the Bayesian model convergence, showing the comparison between the trajectories of fitted model predictions (lighter lines) and the actual observed data (darker line).

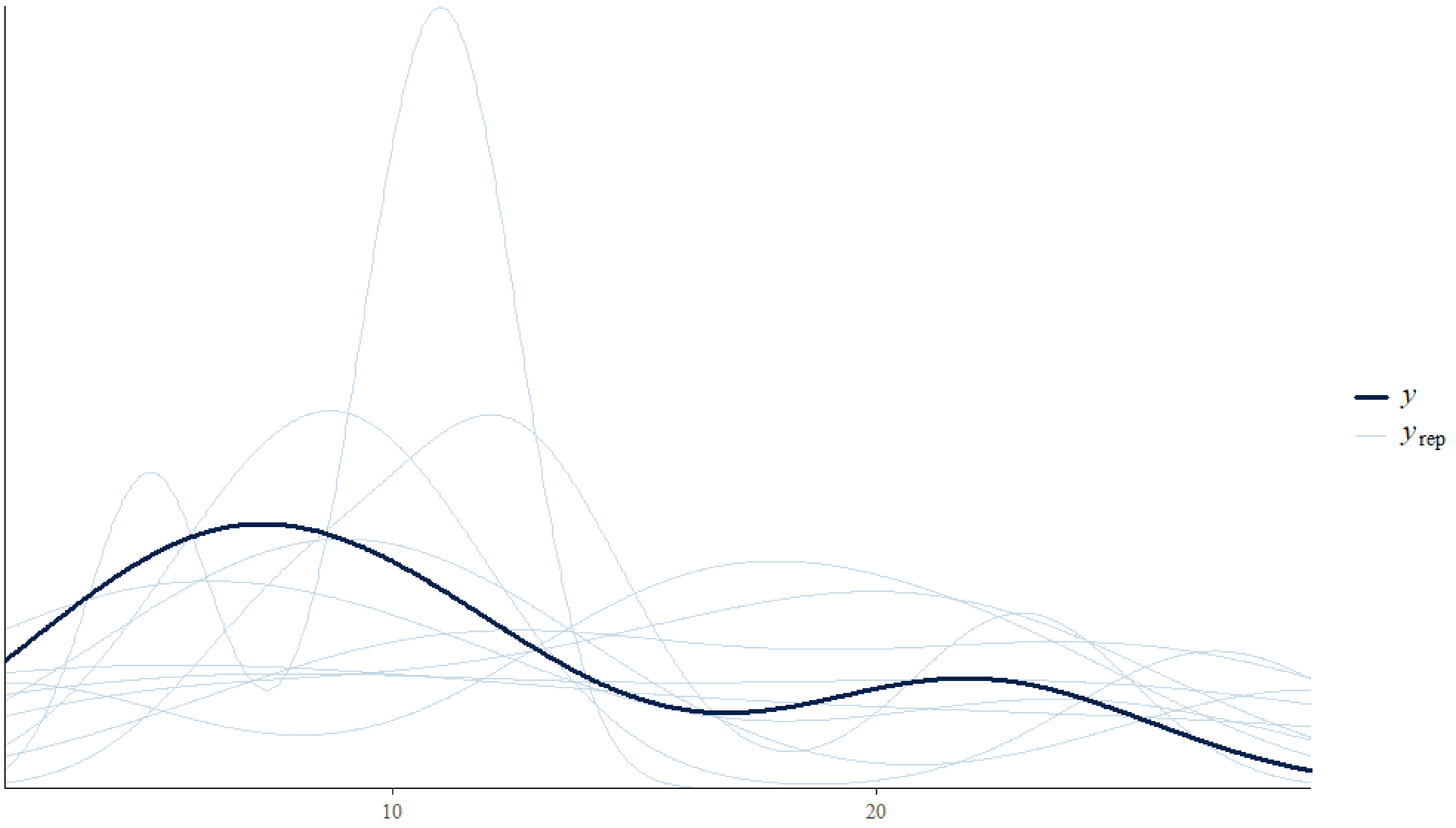

Posterior predictive check (PPcheck) plot for secondary glaucoma. Plot illustrates the Bayesian model convergence, showing the comparison between the trajectories of fitted model predictions (lighter lines) and the actual observed data (darker line).
