# Supplementary material for: Prevalence of glaucoma in Africa: A systematic review and Bayesian meta-analysis
Source: PLoS One. 2025 Aug 14;20(8):e0330567. doi: 10.1371/journal.pone.0330567 (PMC12352844; doi:10.1371/journal.pone.0330567)
Supplement: Attachment — Submitted filename: Peer Review Report.pdf [file pone.0330567.s007.pdf]

Peer Review Report

This manuscript, **"Prevalence of Glaucoma in Africa: A systematic review and Bayesian Meta-Analysis"** presents a timely and methodologically rigorous systematic review and meta-analysis on glaucoma prevalence in Africa. The study addresses a critical gap in understanding regional epidemiological trends and provides valuable insights for public health strategies. However, major revisions are needed to enhance clarity and address methodological limitations & contextual interpretation.

Major Comments

1. **Acronyms and Definitions**

   Acronyms (e.g., PACG, SG) are not consistently defined upon first use in the abstract and introduction. Please define all acronyms (e.g., PACG = Primary Angle-Closure Glaucoma; SG = Secondary Glaucoma) when first introduced to improve readability for non-specialist audiences.

2. **The introduction** is overly verbose, with some paragraphs containing redundant information. Condense lengthy sections (e.g., glaucoma subtypes, risk factors) and ensure a sharper focus on the study's rationale.
3. **Methods**

   Justification for Bayesian Approach: Briefly justify why Bayesian meta-analysis was chosen over frequentist methods.

   Translation Limitations: Acknowledge potential biases/errors from relying on Google Translate for non-English studies. Suggest validation steps (e.g., professional translation for key studies).

   Risk of Bias Assessment: Expand on the results of the Joanna Briggs Institute tool. Specify how many studies were classified as high/moderate/low quality and summarize key methodological weaknesses (e.g., sampling bias).

4. **Results**

   Clarity in Presentation: Ensure tables/figures (e.g., Table 1, Figure 2) are explicitly referenced in the text and described in sufficient detail. For example, clarify what "ISGEO" and "VF/IOP" codes represent in Table 1.

   Subgroup Analyses: Highlight the limited statistical power for PACG/SG due to small study numbers earlier in the results to contextualize their uncertain estimates.

5. **Discussion**
    - **Exploration of Sex Differences**: While estrogen’s role is discussed, consider alternative explanations for male predominance (e.g., occupational exposures, healthcare-seeking behavior).
    - **Geographical Variations**: Expand on potential drivers of higher POAG prevalence in West Africa (e.g., genetic factors, environmental triggers).
    - **Discuss why glaucoma prevalence remains stable** despite global advancements in awareness. Link this to systemic barriers (e.g., under-resourced healthcare systems).
6. **References**: Ensure all in-text citations (e.g., references 42, 43) are included in the reference list. Cross-check numbering to avoid mismatches.

<u>Minor Comments</u>

1. **Abstract**: Clarify that "unclassified glaucoma" refers to cases not categorized into specific subtypes.
2. **Methods:** Specify the search period (e.g., "from inception to January 25, 2025" appears in eligibility criteria but is not explicitly tied to database searches).
3. **Results:** Simplify technical language (e.g., "log-odds estimates") for broader accessibility.
4. **Tables/Figures**:

- Format Table 1 to improve readability (e.g., align columns, use consistent abbreviations).
- In Table 2, define "Half-Normal(0.5)" & other priors in a footnote for clarity

5. **Limitations**: Explicitly mention the exclusion of North/Central African studies as a limitation affecting generalizability.
