# Supplementary material for: Prevalence of glaucoma in Africa: A systematic review and Bayesian meta-analysis
Source: PLoS One. 2025 Aug 14;20(8):e0330567. doi: 10.1371/journal.pone.0330567 (PMC12352844; doi:10.1371/journal.pone.0330567)
Supplement: Attachment — Submitted filename: WS_Review.pdf [file pone.0330567.s008.pdf]

Reviewer Comments for Manuscript ID: PONE-D-25-14594

Title: Prevalence of Glaucoma in Africa: A systematic review and Bayesian meta-analysis

---

General Comments:

This manuscript addresses an important and underexplored topic—the prevalence of glaucoma and its subtypes across African populations—using a systematic review and Bayesian meta-analysis. The methodological approach is sound, and the inclusion of a Bayesian framework is commendable, given the small number of eligible studies and the expected heterogeneity across settings.

However, several methodological clarifications and contextual discussions are needed to enhance the manuscript's scientific interpretability. I offer the following major and minor suggestions for the authors' consideration.

Major Comments:

1. **Justification of Priors in Bayesian Analysis:**

   The authors should provide more detailed justification for the choice of prior distributions, particularly the half-normal distribution with a scale of 0.5 for heterogeneity. Although sensitivity analyses were conducted, a rationale for the selection of informative priors and how they align with previous literature or expert consensus would be valuable.

2. **Scope and Limitations of Included Studies:**

   Only nine population-based studies were included over a two-decade span. This small number significantly limits the generalizability of the findings, especially for PACG and secondary glaucoma, where convergence was not reached in some models. This limitation warrants greater emphasis in the discussion.

3. **Regional Representation and Gaps:**

   The analysis lacks data from North and Central Africa, which limits the applicability of the findings to the entire continent. The authors should highlight this gap more explicitly and suggest how future research can address it.

4. **Risk of Bias Assessment Interpretation:**

The use of the JBI-PCAT tool is appropriate. However, two studies with notable methodological concerns (e.g., recruitment strategy, sample size) were still classified as "low risk." Further clarification of how the scoring was applied and interpreted would improve transparency.

5. **Interpretation of Temporal Trends:**

   The lack of significant change in glaucoma prevalence over time is an important finding. The authors should explore possible explanations (e.g., stagnant healthcare infrastructure, limited uptake of screening programs) and relate this to policy implications more clearly.

Minor Comments:

1. **Language and Grammar:**

   The manuscript would benefit from careful proofreading to correct minor grammatical issues and improve clarity. For instance, the sentence "The title provided in the protocol was amended to suit available evidence" could be made clearer.

2. **Terminology Clarity:**

   Please define the term "glaucoma (unclassified)" clearly in the methods section and use it consistently throughout the text.

3. **Figures and Tables:**

   Consider incorporating brief descriptions or legends of figures directly in the main text. Table 2 should include an explanation of "CrI" (Credible Interval) for readers less familiar with Bayesian statistics.

4. **Discussion on Gender Differences:**

   The discussion on the higher prevalence of glaucoma among males and the potential protective role of estrogen is intriguing. However, these statements are speculative and should be clearly presented as hypotheses supported by limited evidence.

5. **Typographical Errors:**
   - "Cape Coat" should be corrected to "Cape Coast" in the author affiliation.

- The ISGEO/VF/IOP codes in Table 1 should be briefly explained or a legend added to aid interpretation.

6. **Overstatement of Impact (Introduction, Lines 73–76):**

   The statement that this study "holds great potential in providing the needed information for the realization of Sustainable Development Goal 3… and to reduce untimely deaths resulting from blindness from glaucoma" appears to overstate the manuscript's impact. While the study's findings are certainly valuable for public health planning, glaucoma does not directly cause death, and a prevalence estimate alone is unlikely to significantly influence the broader achievement of SDG 3. I recommend rephrasing this sentence to present a more measured and accurate interpretation of the study's contribution.

7. **Undefined Abbreviations in the Abstract:**

   In the abstract, the abbreviations **POAG**, **PACG**, and **SG** are introduced without being defined. While POAG (Primary Open-Angle Glaucoma) and PACG (Primary Angle-Closure Glaucoma) are commonly used terms in ophthalmology, **SG** is not widely recognized and may be ambiguous. I assume it refers to **Secondary Glaucoma**, but this should be clearly stated. Please define all abbreviations at first use in the abstract to ensure clarity for a broader readership.

Recommendation:

With revisions addressing the points above, this manuscript would make a valuable contribution to the literature on glaucoma epidemiology in Africa. I recommend **major revision**.
