# Supplementary material for: Prevalence of glaucoma in Africa: A systematic review and Bayesian meta-analysis
Source: PLoS One. 2025 Aug 14;20(8):e0330567. doi: 10.1371/journal.pone.0330567 (PMC12352844; doi:10.1371/journal.pone.0330567)
Supplement: Attachment — Submitted filename: Final report.pdf [file pone.0330567.s009.pdf]

This manuscript makes a valuable contribution to the literature on glaucoma epidemiology in Africa. The authors addressed comments thoroughly & their revisions align well with the peer review recommendations, making the manuscript stronger and more accessible. The responses were systematic & well-documented, demonstrating a commitment to improving the study's quality. While the manuscript is now robust, a few minor tweaks could polish it further:

- The term "unclassified glaucoma" is clarified but may be confused with "cases not categorized into specific subtypes." Standardizing this phrasing (e.g., always using "unclassified glaucoma") would avoid confusion.
- The forest plots (Figures 2–5) are informative, but adding subtle color differentiation (e.g., for regional subgroups) could enhance interpretability at a glance.
- A few sentences remain slightly verbose (e.g., "The necessity for epidemiological, population-based glaucoma studies in these regions is evident…"). Simplifying to "Population-based studies in these regions are needed…" would tighten the prose.

Final Recommendation:

I recommend acceptance pending these small edits & I look forward to seeing it published.
