# Supplementary material for: Prevalence of glaucoma in Africa: A systematic review and Bayesian meta-analysis
Source: PLoS One. 2025 Aug 14;20(8):e0330567. doi: 10.1371/journal.pone.0330567 (PMC12352844; doi:10.1371/journal.pone.0330567)
Supplement: Attachment — Submitted filename: Response_PONE.pdf [file pone.0330567.s010.pdf]

Dear Editor,

RE: Prevalence of Glaucoma in Africa: A systematic review and Bayesian meta-analysis (Submission ID: PONE-D-25-14594R1).

Thank you for the opportunity to resubmit our manuscript for re-evaluation. Kindly find below a tabulation of point-by-point response to the review comment for your further action.

Comment	Response	Page Number
**Reviewer 1**		
The term "unclassified glaucoma" is clarified but may be confused with "cases not categorized into specific subtypes." Standardizing this phrasing (e.g., always using "unclassified glaucoma") would avoid confusion.	Thank you very much. The phrasing (use of "unclassified glaucoma") has been standardized throughout the manuscript and have been highlighted in red.	Pg 2, ln 34; Pg 9, ln 193; Pg 10, ln 196; Table 2; Pg 15, ln 213, 226, 230, 232; Figure 2; Pg 16, ln 237; Pg 18, ln 297; Pg 313; S2 Fig; S3 Fig
The forest plots (Figures 2–5) are informative, but adding subtle color differentiation (e.g., for regional subgroups) could enhance interpretability at a glance.	Thank you very much for this kind comment. The subtle color differentiation, signifying regional subgroups, have been added to the forest plots.	Figures 2 to 5
A few sentences remain slightly verbose (e.g., "The necessity for epidemiological, population-based glaucoma studies in these regions is evident..."). Simplifying to "Population-based studies in these regions are needed..." would tighten the prose.	Thank you very much. The verbose sentence has been rephrased to tighten up the prose.	Pg 20, ln 345 - 346.
**Editorial Comments**		
If the reviewer comments include a recommendation to cite specific previously published works, please review and evaluate these publications to determine	Thank you very much. The reviewer's comments do not include recommendations to cite previously published works.	

whether they are relevant and should be cited.<br>There is no requirement to cite these works unless the editor has indicated otherwise.	
Please review your reference list to ensure that it is complete and correct.	Thank you very much. The reference list has been reviewed to ensure completeness and accuracy.
If you have cited papers that have been retracted, please include the rationale for doing so in the manuscript text, or remove these references and replace them with relevant current references.	Thank you very much. No retracted paper has been cited within the manuscript.
Any changes to the reference list should be mentioned in the rebuttal letter that accompanies your revised manuscript.	Thank you very much. No change has been made to the reference list.
If you need to cite a retracted article, indicate the article’s retracted status in the References list and also include a citation and full reference for the retraction notice.	Thank you very much. We do not wish to cite a retracted article.
